# Dietary licorice enhances in vivo cadmium detoxification and modulates gut microbial metabolism in mice

**DOI:** 10.1002/imt2.7

**Published:** 2022-03-10

**Authors:** Xin Zheng, Likun Wang, Linhao You, Yong‐Xin Liu, Michael Cohen, Siyu Tian, Wenjun Li, Xiaofang Li

**Affiliations:** ^1^ Hebei Key Laboratory of Soil Ecology, Centre for Agricultural Resources Research, Institute of Genetics and Developmental Biology Chinese Academy of Sciences Shijiazhuang China; ^2^ Laboratory of Molecular Iron Metabolism, The Key Laboratory of Animal Physiology, Biochemistry and Molecular Biology of Hebei Province, College of Life Science Hebei Normal University Shijiazhuang China; ^3^ Institute of Genetics and Developmental Biology, State Key Laboratory of Plant Genomics Chinese Academy of Sciences Beijing China; ^4^ Department of Biology Sonoma State University Rohnert Park California USA; ^5^ University of Chinese Academy of Sciences Beijing China

**Keywords:** cadmium detoxification, gut metabolomics, gut microbiome, licorice, liver, mice

## Abstract

Mass cadmium (Cd) poisoning is a serious health problem in many parts of the world. We propose that dietary intervention can be a practical solution to this problem. This study aimed to identify effective dietary products from traditional Chinese herbs that can detoxify Cd. Five candidate herbal foods with detoxifying potential were selected and subjected to mouse toxicological tests. The chemical composition and dose–response effects of licorice on mouse hepatocytes were determined. Licorice was selected for further tests to examine its effects on growth, tissue Cd accumulation, and gut and liver fitness of mice. The expression of hepatic metallothionein (Mt) genes was quantified in vitro in hepatocytes and in vivo in liver tissues of mice. The results showed that licorice dietary intervention was effective in reducing blood Cd by >50% within 1 month. Cd was also substantially reduced in the heart and lung tissues, but increased 2.1‐fold in the liver. The liver of Cd poisoned mice improved with licorice intervention. Licorice treatment significantly induced Cd accumulation and expression of the *Mt1* gene in hepatic cells both in vitro and in vivo. Licorice intake substantially altered gut microbial structure and enriched *Parabacteroides distasonis*. Omics results showed that licorice improved gut metabolism, particularly the metabolic pathways for glycyrrhizin, bile acids, and amino acids. Dietary licorice effectively reduced mouse blood Cd and had a profound impact on liver and gut fitness. We conclude that herbal licorice can be used as a dietary intervention for mass Cd poisoning.

## Highlights

- We propose that dietary intervention can be a solution to mass Cd poisoning.

- Dietary licorice effectively reduced mouse blood Cd by >50% within a month.

- Licorice intake induced Cd accumulation and *Mt1* expression in mice liver.

- Licorice intake modulated mice gut microbiota and metabolism.

- Overall dietary licorice substantially improved mice fitness under Cd stress.

## Graphical abstract

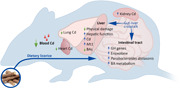

## INTRODUCTION

Excess exposure to heavy metals is inevitable in many parts of the modern world [1]. Metal exposure can be associated with environmental pollution of air, soil, plants, and water. In China, official reports indicate that 19.4% of the total surveyed sites nationwide was contaminated, with cadmium (Cd) accounting for 7% of the pollution [2]. Like soil pollution, heavy metal pollution of the grains in the market was found to be around 14% between 2005 and 2012 [3]. Heavy metal exposure to the population is also of concern in other densely populated countries. In Bangladesh, groundwater arsenic pollution associated with high geological background levels led to the largest mass poisoning in the world [4]. In hotspots of heavy metal pollution, such as mining areas, mass poisoning by chronic or acute heavy metal exposure has been reported for decades.

Recent large‐scale surveys reported that blood Cd/lead poisoning occurred at a concerning rate among children aged 0–6 years in Chinese cities, while in typical mining areas, blood Cd poisoning can be much more serious (Table S1). Pollution control is necessary for reducing Cd exposure; cost‐efficient and effective measures are urgently needed to reduce the impact of Cd on the health of the population, particularly for the residents of mining areas and waste water‐irrigated areas.

Dietary interventions are the most practical approach for large‐scale health interventions [5]. In recent years, dietary interventions have been used for the prevention or treatment of Alzheimer's disease [6], endometriosis [6], weight gain in childhood, acute lymphoblastic leukemia [7], cardiovascular disease [8], depression [9], and type 2 diabetes [10]. For example, tea extracts have been preventing Alzheimer's disease by inhibiting acetylcholinesterase activity [11]. More recently, mannose was found to be a potential dietary treatment for acute urinary tract infections in women [12] and osteoarthritis [13], immunopathology [14], and tumors [15] in mice.

We propose that traditional Chinese dietary herbs may be used for population‐level health interventions associated with mass Cd poisoning. Relative to modern pharmaceuticals, dietary herbs have low toxicity and side effects, and they are inexpensive and easily accessible. For example, licorice is the most widely used Chinese herb, and it works with other medicines to regulate the immune system [16]. Natural products in licorice are mainly used for the treatment of chronic viral hepatitis; it is also widely used for its sweet flavor as a food additive, and it has been approved for use in cosmetics by the United States Food and Drug Administration [17, 18, 19]. One major problem pertaining to the use of such herbs is that Chinese medicine describes herbal pharmaceuticals with a separate syntax specific to the traditional Chinese medicine theory, which cannot be easily translated into modern medical language.

The primary goal of this study was to test the short‐term effects of common Chinese dietary herbs on mouse blood Cd detoxification. Licorice water extract showed a significant positive effect. Therefore, the composition of licorice was determined and its effects on Cd toxicity were examined in terms of Cd tissue concentrations, histopathology, liver functions, and gut microbial diversity and metabolism.

## RESULTS

### Screening for the effects of dietary herbs on acute Cd poison in mice

**Figure 1:**
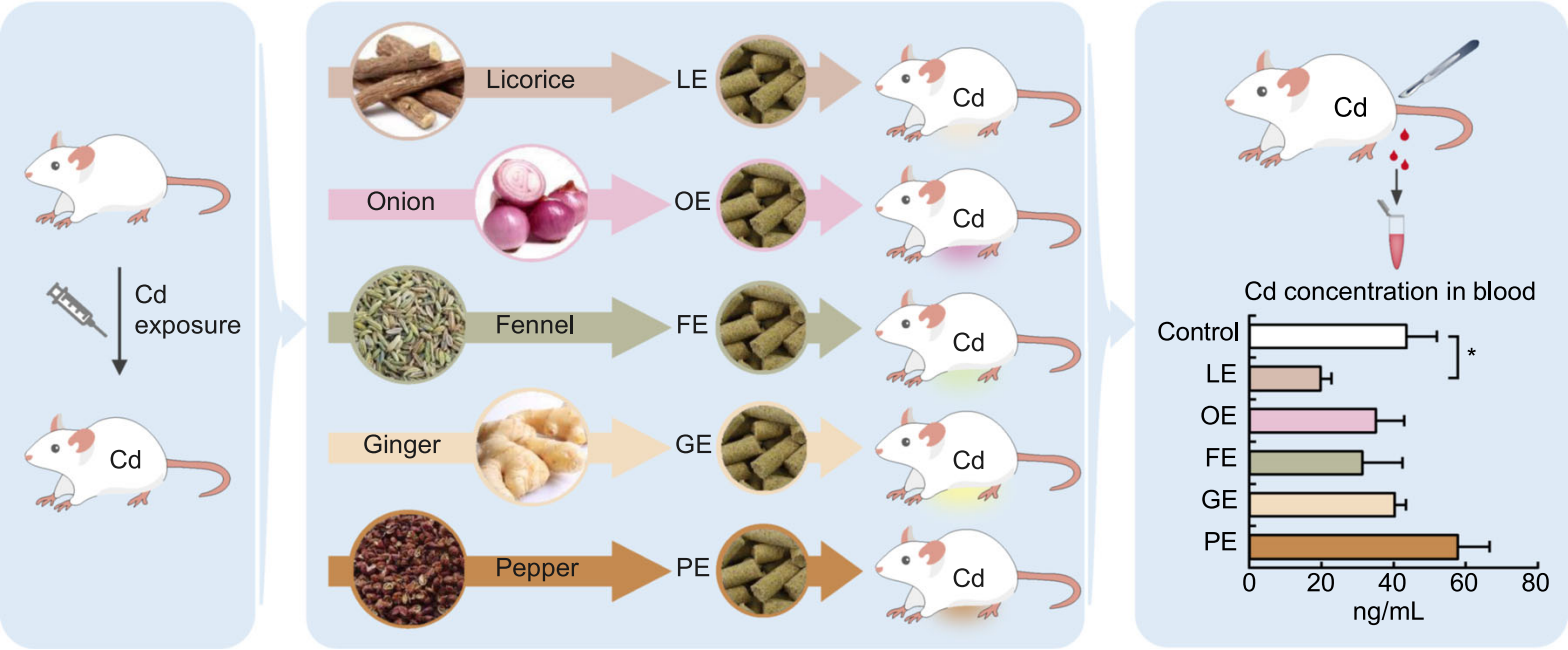
Experimental diagram of the screening of functional dietary herbs for cadmium (Cd) detoxification. Mice were randomly separated into six groups and subjected to intraperitoneal injection of cadmium solution. One‐month dietary interventions were performed by feeding the cadmium‐poisoned mice with herb extract‐supplemented food (FE, fennel extract; GE, ginger extract; LE, licorice extract; OE, onion extract; PE, pepper extract). Blood Cd concentration (mean ± standard deviation (SD), *n* = 3, *P < 0.05, *t* test) was measured to evaluate Cd detoxification by dietary interventions

We tested licorice (LE group), onion (OE group), fennel (FE group), ginger (GE group), and pepper (PE group) by administering them to mice with acute Cd poison (Figure 1). During the 1‐month dietary intervention, each mouse ate 110.97–131.68 g of food, and the body weight increased from 21.03 to 25.78 g, with a weight gain of −0.20 to 3.24 g (Figure S1). No significant (*P* > 0.05, analysis of variance [ANOVA]) difference in food intake or body weight gain was observed between the six groups. There was a decrease in the average level of whole blood Cd content in licorice, onion, fennel, and ginger groups, which was greatest at 54.4% (*P* < 0.05, *t* test) for licorice compared with the control (48.41 ng/mL, Figure 1). These results demonstrated that the dietary licorice was effective in alleviating acute Cd poison in mice.

### Detoxification effect of dietary licorice on acute Cd poising in mice

**Figure 2:**
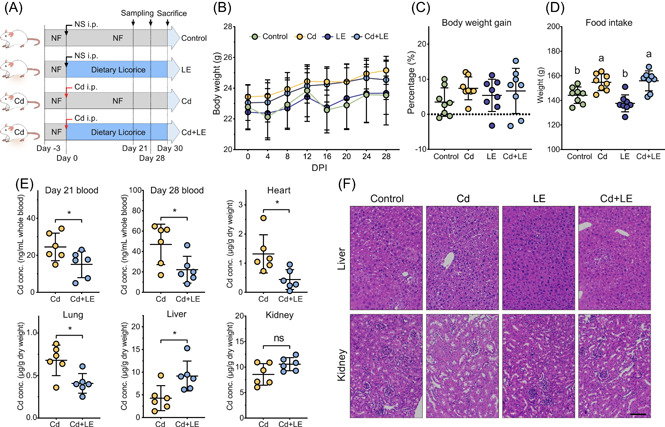
In vivo cadmium detoxification effects of dietary licorice. Mice were randomly separated into four groups (control, LE, Cd, and Cd + LE) and subjected to different treatments following the schematic experimental design and timeline (A). NF, normal food; NS, normal saline; i.p., intraperitoneal injection. (B–D) Body weight change (mean ± SD), body weight gain, and total food intake (mean ± SD) were measured to evaluate survival situation after treatments (*n* = 8, different letters indicate *P* < 0.05, ANOVA). (E) The cadmium concentration in the blood, heart, lung, liver, and kidney was determined (mean ± SD, *n* = 6, *P < 0.05, *t* test). (F) Histopathological assessments of the liver and kidney were conducted, and representative images are shown. Scale bar represents 100 μm. ANOVA, analysis of variance; LE, licorice extract

To further assess the detoxification effect of dietary licorice intervention, a mouse experiment was conducted according to the procedure shown in Figure 2A. A similar pattern of body weight gain was seen among the four treatment groups within 4 weeks of feeding (Figure 2B), with a weight gain range of 3.46%–7.39% on the 28th day (Figure 2C). The body weight of mice exposed to Cd was slightly increased, and there was no significant difference between the two Cd‐free treatments (*P* > 0.05, ANOVA, Figure 2C). Cd exposure significantly increased the food intake by mice compared with the Cd‐free treatments (*P* < 0.05, ANOVA), at a rate of 7.30%–13.32%, whereas no significant change in food uptake was observed in the dietary licorice treatments compared with the control (Figure 2D).

The study results showed that dietary licorice intervention significantly reduced whole blood Cd concentration at Day 28, with a reduction of 52.8% (*P* < 0.05, *t* test, Figure 2E). Meanwhile, licorice intervention significantly reduced Cd concentration in the heart and lung by 66.7% and 40.0% (*P* < 0.05, *t* test), respectively, while it increased the Cd concentration in the liver and kidney (Figure 2E). The Cd concentration in the liver of the Cd + LE treatment group increased 2.1‐fold compared with Cd treatment. The Cd content in the spleen was below the detection limit (data not shown).

Liver histopathology morphologic observation revealed a small amount of edema and no obvious necrosis or inflammatory reaction in the control and LE treatments (Figure 2F). Many hepatocytes had deep nuclear pyknoses, weak cytoplasm staining, and cytoplasmic cavities in the Cd group (Figure 2F). Liver tissue in the Cd + LE group revealed uniform cytoplasm staining and a small amount of edema, but no significant nuclear pyknosis or deep staining in hepatocytes (Figure 2F). In the kidneys, no significant difference was observed among the four groups (Figure 2F). Overall, intraperitoneal injection (i.p.) Cd poisoning led to obvious histopathologic morphologic damage, and LE intervention substantially alleviated these changes.

Further tests were conducted to measure the enzyme activity of aspartate aminotransferase (AST), alanine aminotransferase (ALT), and Γ‐glutamyltranspeptidase (Γ‐GT) in liver tissue, as well as the contents of total bilirubin (TBIL), albumin (ALB), and total bile acid (TBA). Dietary licorice intervention significantly decreased the AST and TBA levels in Cd‐poisoned mice (*P* < 0.05, ANOVA), but it did not change the levels of ALT, Γ‐GT, TBIL, and ALB (Figure S2). Acute Cd poising significantly increased the level of TBA compared with the control group, while no significant difference was observed on the levels of AST, ALT, Γ‐GT, TBIL, and ALB (*P* > 0.05, ANOVA, Figure S2).

### Chemical composition of licorice water extract

**Figure 3:**
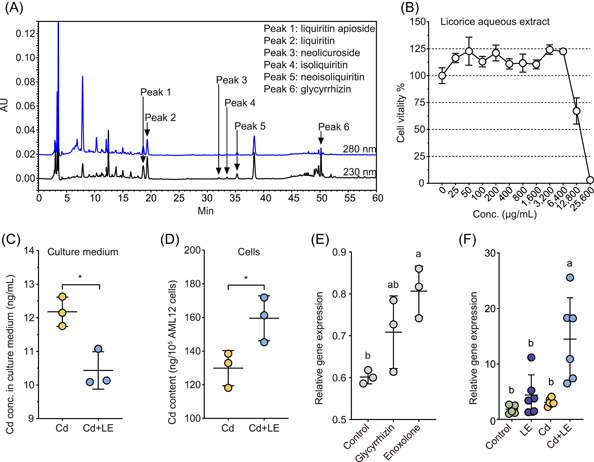
Fingerprint analysis of licorice extract and in vivo test of licorice and cadmium toxicity to hepatocyte. (A) HPLC fingerprints show the main chemical components of the licorice extract. (B) Cell viability test using mouse cell line AML12 against a concentration gradient of licorice extract. Cadmium‐exposed AML12 cells were treated with licorice extract for 24 h, and cadmium content (mean ± SD, *n* = 3, *P < 0.05, *t* test) of (C) medium and (D) cells were determined. (E) Cadmium‐exposed AML12 cells were treated with glycyrrhizin and enoxolone for 12 h, and the expression level of the *Mt1* gene (*n* = 3) was determined using qPCR analysis. (F) Expression level of the *Mt1* gene in liver tissue (*n* = 6) was determined using qPCR analysis after 4 weeks of dietary licorice intervention. HPLC, high‐performance liquid chromatography; LE, licorice extract; qPCR, quantitative polymerase chain reaction

Licorice contains more than 20 triterpenes and 300 flavonoids [19]. The high‐performance liquid chromatography (HPLC) analysis of licorice extract powder used in this study detected peaks of liquiritin apioside (HMDB0037491, Peak 1, at about 18–19 min), neolicuroside (HMDB0040728, Peak 3, at about 32–33 min), isoliquiritin (HMDB0037318, Peak 4, at about 33–34 min), and neoisoliquiritin (HMDB0037317, Peak 5, at about 35–36 min) (Figure 3A). The quantity of the two main ingredients, glycyrrhizin (HMDB0029843) and liquiritin (HMDB0029520), was 17.7 g/100 g and 5.2 g/100 g, respectively (Figure S3).

### Hepatocyte survival tests against acute Cd toxicity and licorice intervention

The dose–response effect of acute Cd poisoning and licorice on hepatic cell viability was examined using the mouse hepatic cell line AML12, under laboratory conditions. The minimum concentration showing the toxic effect of the licorice extract powder on hepatic cell viability was around 12,800 μg/mL. Treatment with 25–6400 μg/mL of the licorice extract led to an increase in cell viability of 10.42%–24.15% (Figure 3B). Likewise, the effects of Cd (CdCl_2_) toxicity on cell viability showed that cell viability began to decrease (91.43% of the control) at a Cd concentration of 12.5 μM (1.41 μg/mL) (Figure S4). On the basis of these results, a concentration of 0.4 μM of Cd was used to pretreat hepatic cells for 12 h to mimic acute Cd poising, followed by 400 μg/mL treatment of licorice extract for 24 h in a fresh medium. A significant reduction in Cd concentration in the medium was observed (*P* < 0.05, *t* test), indicating that licorice extract enhanced Cd immobilization into hepatocytes (Figure 3C). In contrast, Cd concentration in hepatocytes significantly increased with licorice extract treatment (*P* < 0.05, *t* test, Figure 3D). Overall, the in vitro cell line experiment showed results consistent with those of the in vivo experiment.

### Metallothionein gene (*Mt*) expression

The accumulation levels of *Mt* genes mRNA in hepatocyte cells and liver tissues under Cd stress were determined from the mouse experiment described above. Both the main ingredients glycyrrhizin and its intestinal metabolite enoxolone enriched the mRNA of *Mt1*, but not of *Mt2*; enoxolone treatment significantly increased (*P* < 0.05, ANOVA) the mRNA level of *Mt1* by 34.1% in hepatocyte cells (Figures 3E and S4). Likewise, the mRNA level of *Mt1* increased by 7.8‐fold by dietary licorice in liver tissue relative to the control, while no significant effect was detected in the Cd and LE treatment groups (Figure 3F).

### Effect of dietary licorice on gut microbiota

The dietary invention may initially impact gut microbial processes, and microbial metabolism is a key process in the pharmaceutical effect of licorice. To evaluate the response of mouse gut microbial processes to dietary licorice intervention, fresh feces were collected for shotgun metagenomic sequencing and metabolomics analysis.

The shotgun metagenomic sequencing generated 1.06 × 10^9^ optimized clean reads after removing host genomic DNA. A total of 1.60 × 10^11^ bp optimized data were achieved, and 2.51 × 10^6^ contigs with a mean N_50_ of 4,265 bp were obtained (Tables S2 and S3). Gene annotation allowed the identification of 5.01 × 10^6^ open reading frames (ORFs) in 12 samples, and the nonredundant gene set consisted of 7.54 × 10^5^ genes, with a mean length of 696 bp (Table S4). On the basis of the taxonomic annotation of nonredundant genes, 6,602 bacterial species from 106 phyla were identified in this study (Table S5).

**Figure 4:**
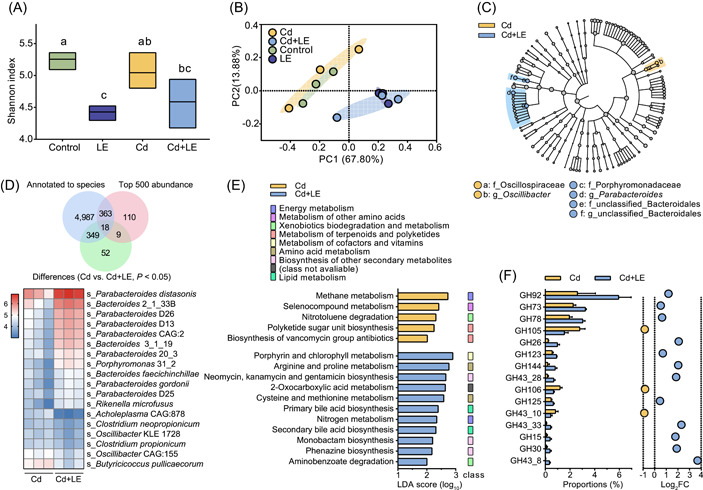
Dietary licorice altered gut microbial composition and function in cadmium‐poisoned mice. (A) Alpha diversity of the gut microbiota in the four treatments as determined using the Shannon index. (B) Principal component analysis of gut metagenomic species profiles among the four treatments. (C) Differentially abundant gut microbial genus between the Cd and Cd + LE treatments through Linear Discriminant Analysis (LDA) Effect Size determination with LDA value > 3.0. (D) Principal differentially abundant gut microbial species between the Cd and Cd + LE treatments through screening the overlaps among microbiota annotated to species, top 500 abundant microbes, and differentially abundant gut microorganisms between the Cd and Cd + LE treatments; abundance of the 18 selected species in the Cd and Cd + LE treatment groups was visualized using a heatmap. (E) LDA for KEGG metabolic pathways in the Cd and Cd + LE treatment groups. (F) Differentially abundant carbohydrate‐active enzyme (CAZy) genes between the Cd and Cd + LE treatment groups annotated by the CAZy database. KEGG, Kyoto Encyclopedia of Genes and Genomes; LE, licorice extract

Alpha diversity analysis showed that gut bacterial diversity was significantly reduced by dietary licorice intake, but not acute Cd i.p. exposure (Figure 4A and Tables S5 and S6). Principal coordinates analysis (PCoA) revealed that dietary licorice separated the gut microbiota into two clusters: Cd and the control cluster; and LE and Cd + LE cluster (Figure 4B). At the phylum level, dietary licorice intake enriched *Bacteroidetes* and reduced the abundance of *Firmicutes*; at the genus level, dietary licorice intake mainly increased the abundance of *Parabacteroides*, *Prevotella*, and unclassified *Bacteroidales*, and reduced the abundance of *Clostridium*, and unclassified *Lachnospiraceae* and *Firmicutes* (Figure S5).

Linear discriminant analysis (LDA) Effect Size results showed that the outstanding gut microbes (LDA score > 3.0) in the Cd + LE treatment included *Porphyromonadaceae*, *Parabacteroides*, and two unclassified *Bacteroidales*, while the typical gut microbes in Cd treatment were *Oscillospiraceae* and *Oscillibacter* (Figure 4C). These results were similar to those obtained when the control was compared with LE treatment (Figure S6). Cd treatment did not lead to a substantial change in gut microbial composition as compared with the control (Figure S7). A total of 18 key well‐annotated species were selected in the overlap species set (Figure 4D). Dietary licorice primarily enriched *Parabacteroides* species, among which *Parabacteroides distasonis* was the most abundant (Figure 4D).

LDA for Kyoto Encyclopedia of Genes and Genomes (KEGG) metabolic pathways showed that dietary licorice principally enriched 11 KEGG metabolic pathways (*P* < 0.05), including porphyrin metabolism, amino acid metabolism (arginine‐, proline‐, cysteine‐, and methionine‐related pathways), antibiotic metabolism (neomycin‐, kanamycin‐, gentamicin‐, and streptomycin‐related pathways), and bile acid metabolism (Figure 4E). The distinct KEGG metabolic pathways of Cd treatment were methane metabolism, selenocompound metabolism, nitrotoluene degradation, polyketide sugar unit biosynthesis, and biosynthesis of vancomycin group antibiotics (Figure 4E).

The differentially abundant carbohydrate‐active enzyme (CAZy) genes annotated by the CAZy database were further analyzed. The class most regulated by dietary licorice was glycoside hydrolases (GH), in which GH92, GH73, and GH78 showed a significant increase in abundance (Figure 4F). GH15 and GH30, which include glucosidase and glucuronidase, showed a 3.43‐ and 3.72‐fold increase in abundance, respectively. There were 10 families, 9 families, 8 families, 5 families, and 1 family showing significantly differential abundance in the polysaccharide lyases (PL), carbohydrate‐binding modules (CBM), glycosyl transferases (GT), carbohydrate esterases (CE), and auxiliary activities (AA) families, respectively (Figure S8).

### Effect of dietary licorice on gut metabolites

Metagenomic analysis showed that the gut microbial community and its metabolic function were changed dramatically, driven by dietary licorice. To investigate whether those changes influenced intestinal metabolism, fresh feces were subjected to metabolomics analysis.

**Figure 5:**
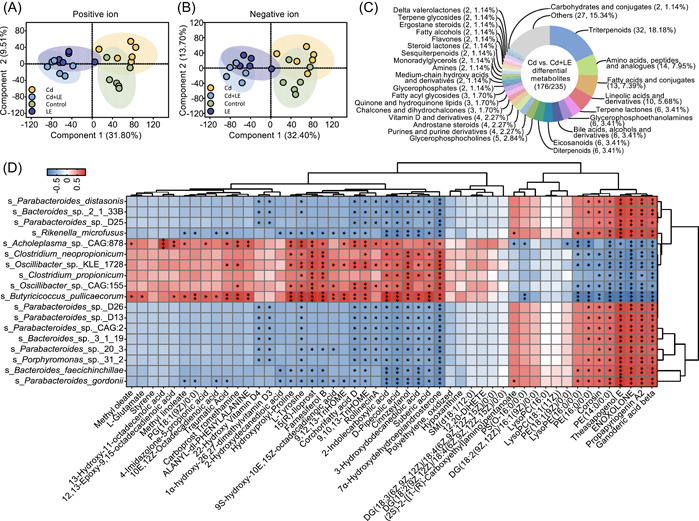
Metabolite variation between Cd and Cd + LE groups, and the correlation between gut metabolites and microbes that discriminated the Cd + LE group from the Cd group. Partial least‐squares discriminant analysis (PLS‐DA) of positive (A) and negative (B) ion metabolites. (C) Differentially abundant metabolites between the Cd group and the Cd + LE group. (D) Correlation between the 18 key differentially abundant microbial species and top 50 differentially abundant metabolites in the Cd and Cd + LE groups. Positive correlation is displayed in red, while negative is marked with blue color. *P < 0.05, **P < 0.01, and ***P < 0.001. LE, licorice extract

Liquid chromatography–mass spectrometry (LC‐MS) label‐free metabolomics analysis identified 12,430 positive ions and 11,750 negative ions, among which 636 were identified based on their molecular weight and 547 were annotated by human metabolome database (HMDB; Table S7). Partial least‐squares discriminant analysis (PLS‐DA) to both positive and negative ion showed that dietary licorice intervention had significant impacts on metabolites in mouse feces, compared with licorice‐free treatments; in contrast, slight metabolite composition changes were observed in the feces of mice exposed to Cd compared with the Cd‐free treatments (Figure 5A,B), which was consistent with the PCoA result of the gut metagenome. Further, a total of 235 significantly differential metabolites were obtained between the Cd and Cd + LE treatments, among which 176 were successfully annotated by HMDB (Figure 5C). Terpenoids, including monoterpenes, diterpenes, and triterpenes (32), accounted for the largest proportion of the 235 differential metabolites (Figure 5C). Additionally, flavonoids and quinones were also observed in the 235 differential metabolites (Figure 5C). The categories of amino acids (14), fatty acids (13), lineolic acids (10), terpene lactones (6), glycerophosphoethanolamines (6), bile acids and derivatives (6), and eicosanoids (6) were also among the top differential metabolites (Figure 5C).

Furthermore, the above‐mentioned 18 key differential gut microbial species correlated with the top 50 abundant differential metabolites. Among the 50 metabolites, several significant correlations (*P* < 0.05) were detected (Figure 5D). In general, two clusters of metabolites showed significant correlations with almost all 18 selected microbial species, but with a contrasting pattern of negative/positive correlations. One cluster included five licorice‐derived triterpenes (Corosin, Theasapogenol E, ENOXOLONE, Propapyriogenin A2, and Ganoderic acid beta) and three ethanolamine phosphates (PE [16:0/0:0], LysoPE [16:0/0:0], and PE [14:0/0:0]) (Figure 5D). Another cluster included corchorifatty acid D, 9,10,13‐TriHOME, rollinecin A, 2‐indolecarboxylic acid, d‐pipecolic acid, cinnzeylanol, 3‐hydroxydodecanedioic acid, suberic acid, and 7α‐hydroxydehydroepiandrosterone (7α‐OH‐DHEA) (Figure 5D). l‐tyrosine, which is a potential biomarker for long‐term exposure to environmental Cd [20, 21], showed significantly negative correlations with almost all the 11 enriched microbial species.

## DISCUSSION

### Cadmium‐detoxifying effects of dietary licorice

The dietary herbs selected in this study are commonly used as foods or flavors in China and have been used as herbal medicines since ancient times. Licorice, onion, and ginger are generally used for “Jie Du,” which literately means “detoxification” (“Pharmacopoeia of the People's Republic of China” and “Chinese Materia Medica”). Only licorice showed significant Cd‐detoxifying effects in mice within 1 month (Figure 1). From a viewpoint of modern medicine, “Jie Du” is a broad concept that covers detoxification of toxic substances, such as venom and phytotoxins, as well as alleviation of a variety of inflammatory diseases, such as sore throat, fever, and oral ulcers. Our results indicate that traditional “Jie Du” herbs are not equally effective in alleviating mouse Cd toxicity, and we thus suggest that the traditional concept of “Jie Du” cannot be simply used for metal detoxification in modern medicine. A number of studies have demonstrated that licorice showed pharmacological effects in various hepatic diseases [18, 22, 23, 24, 25, 26] and heavy metal poisoning [27]. However, this is the first study to investigate its effects on Cd elimination from multiple tissues of experimental mice. Cd bioaccumulation in tissues of mice is dose‐dependent, and excess Cd exposure may lead to rapid overload in these tissues [28, 29]. In this study, we used i.p. Cd exposure for rapid generation of Cd‐poisoned mouse models, which is an efficient approach for toxicological studies [30]. The Cd dose was selected based on literature research and pilot experiment, which was comparable to the ones used in previous studies and was able to cause moderate toxicity, with no obvious tissue adhesion in the liver or significant reduction in body weight or food intake (results not shown). In mammals, blood flow and circulation centers in the substance interchange and nutrient delivery play an important role in Cd transport and redistribution among different tissues [28]. Several studies have verified the strong positive correlation between blood Cd content and body Cd burden [31, 32, 33]. Thus, blood Cd content is a reasonable indicator of the level of Cd toxicity. Using blood Cd as an indicator, dietary licorice was the only one among the five tested herbs that significantly reduced blood Cd content within 1 month (Figure 1).

We found that dietary licorice promoted Cd immobilization in the liver, which may be responsible for Cd elimination in the blood, heart, and lung. Studies over the past decades have reported that the main Cd reservoirs in the body are the liver and kidney; the majority of Cd ions were first captured by MTs to form Cd‐MT complexes in hepatocytes [28]. A small amount of Cd‐MT can be released into the blood from the liver, and slowly to the kidneys, where Cd is eliminated mainly in the urine [28]. Therefore, Cd accumulation in the liver and kidney is indispensable in mouse Cd detoxification in vivo. Our mouse experiments showed that dietary licorice increased Cd accumulation in the liver and kidney (Figure 2), and our in vitro results demonstrated that licorice extract maintained Cd immobilization in hepatocytes (Figure 3). In parallel, the *Mt1* gene was substantially induced by licorice extract, particularly enoxolone (Figure 3). The significant effect of enoxolone on Mt gene induction in the mouse liver is consistent with previous findings that the pharmaceutical effect of licorice relies on its metabolism by gut microbes from glycyrrhizin to enoxolone [34, 35]. Evidence from histopathological imaging supports the idea that the dose of licorice extract used in this study did not cause substantial extra damage to the liver under i.p. Cd poisoning (Figure 2), although moderate negative impacts of licorice on health have been reported in recent years [36]. In fact, licorice extract mitigated the Cd‐induced changes of ATS and TBA indices (Figure S2), without affecting the food intake and body weight of mice (Figure 2). Taken together, these results suggest beneficial effects of dietary licorice on mouse fitness after Cd exposure.

### Dietary licorice modulates mouse gut microbiota

The pharmacological components of licorice include triterpenes, flavonoids, as well as various polysaccharides [19]. HPLC analysis showed that the commonly known components of licorice were detected in the aqueous extract used in the current study (Figure 3), and glycyrrhizin had the highest abundance. Glycyrrhizin is known to have pharmacological effects mainly after being converted by gut microflora to enoxolone [34, 35], which was identified with high abundance in the fecal metabolome (Figure 5). In recent years, gut microbiota was found to play important roles in the host metabolism of herbal components [37], including the microbial deglycosylation of saponins [38]. Indeed, we observed significant increases in the abundance of several GH families in the metagenomic assemblages, including GH15 and GH30 (Figure S2), which may contribute to the deglycosylation of glycyrrhizin. Significant changes in the abundance of other CAZy families were also observed (Figure S8). Furthermore, the abundance of key enriched gut bacteria was positively correlated with five triterpenoids derived from licorice (Figure 5D). This suggests that mouse gut microbiota may have a substantial impact on the metabolism and pharmaceutical effects of dietary licorice.

Consistent with previous studies, dietary licorice showed profound effects on the gut microbial community structure and metabolism (Figures 4 and 5). Unexpectedly, the use of Cd alone did not cause a significant shift in the gut microbial structure or fecal metabolome (Figures 4, S4, and S6), whereas obvious toxicological effects were induced by Cd exposure in terms of liver histopathological changes. Several studies have suggested that gut microbiota is sensitive to Cd exposure through administration and oral intake [30]. The relatively low dosage of Cd (0.3 mg/kg) may partly account for the little effect on mouse gut microbial community structure. Nonetheless, *Lactobacillaceae* and *Lactobacillus*, many of which are generally believed to be associated with health [39, 40, 41], were suppressed by Cd poisoning compared with the control (Figure S7). In companion with a reduced diversity induced by dietary licorice (Figure 4A), some prominent changes in the gut microbial community structure, which may have beneficial effects on mouse health, were also observed. These changes included the enrichment of *Bacteroides* and *Parabacteroides* genera [42, 43, 44, 45], particularly *P. distasonis* (Figure 3C). *P. distasonis* showed a negative correlation with obesity, nonalcoholic fatty liver disease, diabetes, inflammatory bowel disease, and multiple sclerosis [44, 46, 47, 48, 49, 50]. Another bacterium, *Bacteroides gordonii*, was enriched by the dietary licorice and has been reported to be negatively correlated with obesity and rheumatoid arthritis [51, 52]. Nevertheless, it is still unclear whether the microbiome changes induced by licorice intake are a common health effect of licorice, though the current results indicate that dietary licorice may dedicatedly reduce blood Cd and alleviate physical damages of Cd to the host.

### Dietary licorice fine tunes the mouse intestinal metabolism

**Figure 6:**
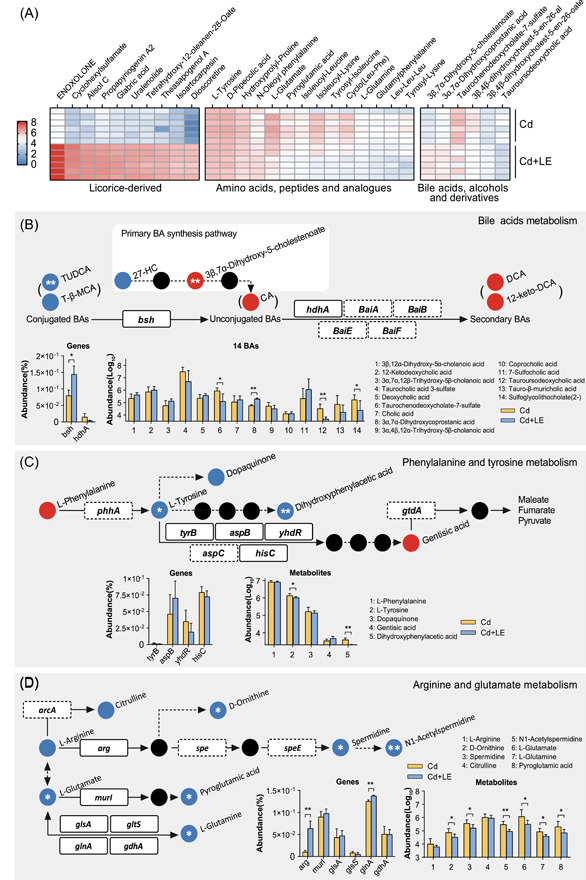
Key metabolites and potential metabolic pathways of gut microbiota associated with the dietary licorice intervention. (A) A heatmap illustrating the metabolites that discriminated Cd + LE from Cd groups (*P* < 0.05). Top 10 abundant licorice‐derived metabolites and gut metabolites are shown. (B–D) Representative metabolites, relative enzyme‐encoding genes, and involved metabolic pathways. The pathways were constructed based on the KEGG metabolic maps. Metabolites are indicated as red (enriched in the Cd + LE group), blue (enriched in the Cd group), or black (none detected) balls. Identified microbial enzyme‐encoding genes are represented in boxes (the dashed one means poor abundance). The dashed arrow indicates the potential metabolic process without detection of relevant enzyme‐encoding genes. Relative abundance of the involved metabolites (mean ± SD, *n* = 6, *P < 0.05, **P < 0.01, *t* test) and genes (mean ± SD, *n* = 3, *P < 0.05, **P < 0.01, *t* test) are shown. 12‐keto‐DCA, 12‐ketodeoxycholic acid; DCA, deoxycholic acid; KEGG, Kyoto Encyclopedia of Genes and Genomes; LE, licorice extract; TUDCA, tauroursodeoxycholic acid; T‐β‐MCA, tauro‐β‐muricholic acid

Metabolomics evidence supports the beneficial effects of dietary licorice on mouse fitness. Fecal metabolites showed a strong modulation by dietary licorice, but not i.p. Cd exposure (Figure 5A,B), which was consistent with the results of the metagenomic analysis. Several metabolites (e.g., l‐glutamine, l‐tyrosine, and xanthine), whose abundance was significantly reduced in the Cd + LE treatment group (Figure 6A), have been identified as potential biomarkers for the long‐term exposure of environmental Cd [20, 21]. A significant negative correlation was observed between 7α‐OH‐DHEA, d‐pipecolic acid, and 3‐hydroxydodecanedioic acid and the key enriched gut germs (Figure 5D), which may be associated with chronic inflammation, chronic liver disease, and peroxisomal disorders [53, 54, 55, 56, 57, 58]. Considering this evidence, we conclude that dietary licorice intervention had a positive effect on mouse fitness under Cd stress, in terms of Cd elimination from blood, lung, and heart, as well as liver functions, liver histopathological alleviation, gut microbial composition, and fecal metabolite profiles.

The high‐throughput annotation of gut metagenomes and metabolomes enables the integration of gut microbial metabolic pathways in response to Cd exposure and dietary licorice intervention. Some prominent metabolic pathways were detected, including those of bile acids, amino acid, fatty acids, lineolic acids, glycerophosphoethanolamines, and eicosanoids (Figure 5C). Notably, 14 bile acids and derivatives were identified in the metabolomes (Figure 6B), of which four were found to be responsive to the licorice intervention. Two conjugated bile acids, tauroursodeoxycholic acid (TUDCA) and tauro‐β‐muricholic acid (T‐β‐MCA), were actively metabolized to secondary bile acids, typically 12‐ketodeoxycholic acid (12‐keto‐DCA) and deoxycholic acid (DCA), possibly via the cholate deconjugation gene *bsh* (K01442), in the Cd + LE treatment group. The enrichment of *Parabacteroides* and *Bacteroides* species (Figure 4C,D), which possess bile acids metabolic activities [44, 59, 60], in the Cd + LE group may partly account for these changes. Unconjugated bile acids were generally believed to be potent activators of the TGR5 signaling pathway [59]. TGR5 regulates energy metabolism and induces the differentiation of enteroendocrine cells [59, 60, 61, 62]. TUDCA and T‐β‐MCA, antagonists of the farnesoid X receptor (FXR) signaling pathway [63, 64, 65, 66, 67, 68], showed downregulation in abundance, implying the regulation of the FXR pathway and bile acids synthesis in the liver. FXR functions as a regulator of bile acid synthesis and plays a positive role in inflammation and immunity in the liver and gastrointestinal tract [59, 60, 61]. Indeed, the contents of TBAs in the liver were reduced by the dietary licorice (Figure S2F). We speculate that the modulation of bile acid metabolism may be an important effect of the dietary licorice intervention and may have a profound impact on mouse fitness under Cd stress.

Amino acid metabolism was also affected by licorice intake (Figure 6C,D). Because no changes in food intake were observed between the Cd group and the Cd + LE group (Figure 2D), it is possible that dietary licorice promoted the fermentation of the amino acids, such as tyrosine and glutamate, into short‐chain fatty acids. We detected a variety of enzyme‐coding genes for amino acid metabolism, particularly *arg* (K01476) and *glnA* (K01915), which were enriched in the Cd + LE group (Figure 6D). In addition to bile acid and amino acid metabolism, many other metabolic pathways were also affected by licorice treatment (Figure S9). The associations of most pathways with mouse fitness under Cd stress remain to be explored.

**Figure 7:**
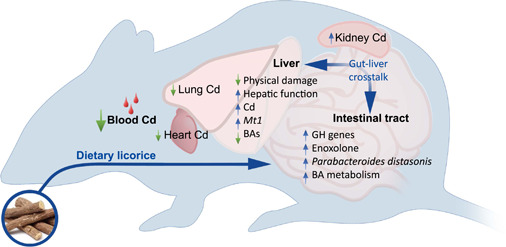
A schematic diagram illustrating the proposed mechanism of dietary licorice intervention on health under Cd stress. Dietary licorice intake alters the diversity of the mouse gut microbiome, increases the abundance of *Parabacteroides distasonis*, and enriches glycoside hydrolases (GH) genes and bile acid metabolic pathway. Glycyrrhizin is actively converted by gut microflora into enoxolone. Enoxolone is transferred into the liver and alleviates the liver damage caused by Cd in terms of histopathological analysis and hepatic function, by sharply inducing *Mt1* gene expression. The liver and gut may have interaction via bile acids, which affects the gut microbiome structure. As a result of Cd accumulation in the liver, Cd accumulation in other tissues was reduced

Dietary licorice is effective in improving mouse fitness under Cd stress (Figure 7). Licorice has great potential to be used for dietary intervention for mass Cd poisoning.

## METHODS

### Experimental design

We searched classic Chinese herbal books and identified five dietary herbs, namely, *Glycyrrhiza uralensis* Fisch. (Licorice, L), *Allium cepa* L. (Onion, O), *Zingiber officinale* Roscoe (Ginger, G), *Zanthoxylum bungeanum* Maxim. (Sichuan pepper, P), and *Foeniculum vuLgare* Mill. (Fennel, F), which have been used as medicinal herbs for generic detoxification since ancient times. They were subjected to animal toxicological tests using laboratory mice (Figure 1); positive results were obtained for licorice. Licorice was further tested via animal tests, where tissue Cd determination (whole blood, heart, lung, liver, kidney, and spleen), histopathology imaging, liver function tests, shotgun gut metagenomic sequencing, and feces metabolomics analysis were performed. Overall, the aim of these experiments was to screen and test whether dietary herbs can alleviate acute Cd toxicity and enhance gut fitness.

### Experimental animals

Male BALB/c mice, weighing approximately 22 g, were purchased from Hebei Laboratory Animal Centre. The mice were maintained in separate cages in a clean animal house at 21 ± 2°C and under a 12‐h day/night cycle. The study was approved by the Animal Care and Use Committee of the Hebei Science and Technical Bureau, China, and performed in accordance with the guidelines of the Chinese Association for Laboratory Animal Sciences.

### Dietary herbs

Standard laboratory mouse food was purchased from Hebei Laboratory Animal Centre. Licorice (L), onion (O), ginger (G), pepper (P), and fennel (F) were purchased from the local market (Shijiazhuang, China). To prepare food containing the dietary herbs, licorice roots and rhizomes, onion bulbs, ginger rhizomes, pepper pericarp, and fennel nuts were cleaned, ground, and subjected to distilled water extraction three times. The tripartite solutions were mixed, filtered, and subjected to powder spray drying with a concentration density of 1.20. The foods containing the dietary herbs were made by mixing the aqueous extract powders (LE, OE, GE, PE, and FE) with the standard food at a ratio of 1:9 and extrusion molding.

### Screening for effective dietary herbs

Eighteen mice were randomized into six groups for dietary herb interventions (control, LE, OE, GE, PE, and FE). All the mice were allowed to adapt to the animal house with a standard food supply for 3 days before the injection. At Day 0, mice were intraperitoneally (i.p.) injected with normal saline (NS) plus Cd (CdCl_2_; 0.3 mg/kg) to induce acute Cd poisoning. Mice were provided free access to water and food (standard or containing dietary herb). At Day 30, whole blood samples (*n* = 3) were obtained from the caudal vein to determine the Cd content.

### Pharmaceutical assessment of the dietary licorice extract

Twenty‐four mice were prepared as mentioned previously, and randomly divided into four groups: control, LE, Cd, and Cd + LE. On Day 0, mice were treated with NS (100 μL; 0.9% NaCl; i.p.) for control and LE groups and Cd (CdCl_2_; 0.3 mg/kg; i.p.) for Cd and Cd + LE groups. Control and Cd mice had free access to water and standard food, while LE and Cd + LE mice had free access to water and licorice‐containing food. The body weight and food consumption of mice were measured every 3 days. Fresh feces (*n* = 6) were harvested on Day 28 for metagenomics and metabolomic analysis. Blood samples (*n* = 6) were obtained twice from the caudal vein on Days 21 and 28 for the determination of Cd content. On Day 30, all of the mice were anesthetized using sodium pentobarbital (40 mg/kg; i.p.) and subjected to perfusion with ice‐cold saline solution to remove body blood. The main organs were harvested for Cd content determination, histopathological analysis, and other tests.

### Cd content in tissues

Tissue samples were dried, grounded, and digested using nitric acid and a microwave digester (Multiwave PRO; Anton‐Paar), according to the manufacturer's instructions. Cd concentration of the digests was determined by graphite atomic absorption spectrometry (TAS‐990; Persee) with CRM Laver as the standard material (GWB10023; certified by Institute of Geophysical and Geochemical Exploration).

### Histopathological analysis and hepatic function

Hematoxylin and eosin staining was used to evaluate the histopathology of the liver, lung, kidney, heart, and spleen. Briefly, samples were fixed, dehydrated, paraffin‐embedded, and sliced into 30‐μm sections. These sections were stained with hematoxylin and eosin, followed by dehydration. The tissue morphology was observed using a light microscope. Liver samples were examined to evaluate the effect of Cd and Glycyrrhiza on live functions. Briefly, liver tissues were well ground with NS solution, and the supernatant was obtained by centrifuging the homogenate at 4000*g* for 10 min. The total protein concentration in the supernatant samples was determined using Pierce BCA protein assay kit (Thermo Fisher Scientific). The ALT, AST, and Γ‐GT levels in the supernatant samples were measured using commercial test kits, according to the manufacture's protocols (Changchun Huili Biotech Co. Ltd.). The final indices were calculated by dividing the measured values by the total protein concentration for each sample (*n* = 6).

### Fingerprint profile of licorice extract and quantitative determination of glycyrrhizin and liquiritin

The licorice aqueous extract powder was thoroughly dissolved with 70% ethanol and filtered through 0.22‐μm Millipore filters. The treated solution was subjected to HPLC analysis using a Waters 2695–2996 liquid chromatography system (Waters Corporation). The licorice components were separated by chromatography using an Ultimate XB‐C_18_ column (5 μm; 4.6 × 250 mm; FX‐014; Welch Materials Inc.) at 35°C. The mobile phase consisted of (A) acetonitrile and (B) 0.1% phosphoric acid, with a flow rate of 1.0 mL/min. We injected 10 μL of the sample for 60 min. The detection wavelengths were 230 and 280 nm. Appropriate quantities of glycyrrhizin and liquiritin were weighed and dissolved in 70% ethanol. HPLC analysis described previously was performed for the standard solutions with different concentrations. The areas of each peak at 237 nm detection wavelength were obtained to draw standard curves. The same HPLC analysis was performed for the test sample, and contents of glycyrrhizin and liquiritin in the licorice aqueous extract powder were calculated.

### Dose–response tests of mice hepatocytes in response to Cd and licorice

Mouse hepatocyte AML12 was cultured in Dulbecco's modified Eagle's medium (DMEM) plus 10% fetal bovine serum (FBS), and placed in an incubator at 37°C with 5% CO_2_ and 99% humidity. For the viability test, AML12 cells were seeded in 96‐well plates, and incubated with Cd (CdCl_2_; 0.049, 0.098, 0.0195, 0.391, 0.781, 1.563, 3.125, 6.250, 12.500, 25.000, and 50.000 μM) or licorice extract solution (25, 50, 100, 200, 400, 800, 1600, 3200, 6400, 12,800, and 25,600 μg/mL) for 24 h. Cell viability (*n* = 8) was measured using a cell counting kit‐8 assay (Dojindo Laboratories). For the Cd detoxification test, AML12 cells were seeded in 6‐well plates, and incubated with 0.4 μM Cd solution for 12 h; then, the culture medium was replaced and the Cd‐treated cells were incubated with 400 μg/mL licorice solution for 24 h. Cells and culture media (*n* = 3) were harvested for Cd content determination described above.

### Gene expression analysis

Mouse hepatocyte AML12 was cultured as described above. Cells were incubated with 0.4 μM Cd solution for 12 h; then, the culture medium was replaced and the Cd‐treated cells were incubated with 57.15 μg/mL licorice extract (containing 12.5 μM glycyrrhizin), 12.5 μM glycyrrhizin, and 12.5 μM enoxolone for 12 h. Cells were then harvested. Total RNA was extracted from the cells (*n* = 3) and mouse liver tissue (*n* = 6) using an Ultrapure RNA kit (CWBIO), according to the manufacturer's protocol. A total of 2 μg RNA was used for reverse transcription using a FastQuant RT Kit (Tiangen Biotech [Beijing] Co. Ltd.), according to the manufacturer's instruction. The quantitative polymerase chain reaction (PCR) was performed using a Bio‐Rad CFX Connect Real‐Time PCR Detection System and SYBR Green SuperReal PreMix Plus (Tiangen Biotech [Beijing] Co. Ltd.). The thermo condition was set at 95°C for 15 min, followed by 40 cycles at 95°C for 10 s and at 61°C for 30 s. Mouse *beta‐actin* gene (GenBank ID: 11461) was used as the internal reference. Relative gene expression levels were detected using the $2^{‐∆∆C_{t}}$ method. The primer sequences were 5′‐CTCCGTAGCTCCAGCTTCAC‐3′ and 5′‐AGGAGCAGCAGCTCTTCTTG‐3′ for *Mt1* (GenBank ID: 17748), 5′‐TGTGCTGGCCATATCCCTTG‐3′ and 5′‐GCGGAGAGTATTGGGTCGAG‐3′ for *Mt2* (GenBank ID: 17750), and 5′‐AGGCCCAGAGCAAGAGAGGTA‐3′ and 5′‐TCTCCATGTCGTCCCAGTTG‐3′ for *beta‐actin*.

### Feces DNA extraction, library construction, and metagenomic sequencing

Total genomic DNA of mouse fecal samples was extracted using a QIAamp PowerFecal DNA Kit (QIAGEN), following the manufacturer's instructions. The concentration and purity of extracted DNA were determined using TBS‐380 and NanoDrop2000, respectively. DNA quality was checked on 1% agarose gel. DNA extract was fragmented to an average size of about 400 bp using Covaris M220 (Gene Co. Ltd.) for paired‐end library construction. A paired‐end library was constructed using NEXTFLEX Rapid DNA‐Seq (Bio Scientific). Adapters containing the full complement of sequencing primer hybridization sites were ligated to the blunt end of the fragments. Paired‐end sequencing was performed using Illumina NovaSeq/Hiseq Xten (Illumina Inc.) at Majorbio Bio‐Pharm Technology Co. Ltd., using NovaSeq Reagent Kits/HiSeq X Reagent Kits, according to the manufacturer's instructions. Sequence data associated with this project have been deposited in the NCBI Short Read Archive database.

### Sequence quality control (QC) and metagenomic assembly

The data analyses refer to the steps of EasyMetagenome 1.10 pipeline [69]. Paired‐end Illumina reads were trimmed of adapters, and low‐quality reads (length < 50 bp or quality value < 20 or having N bases) were removed using fastp [70] (version 0.20.0). Reads were aligned to the mouse genome using BWA [71] (version 0.7.9a; http://bio-bwa.sourceforge.net), and any hit associated with the reads and their mated reads were removed. Metagenomics data were assembled using MEGAHIT [72] (version 1.1.2; https://github.com/voutcn/megahit), which uses succinct de Bruijn graphs. Contigs with lengths of 300 bp or more were selected for the final assembling result, and the contigs were used for gene prediction and annotation.

### Gene prediction, taxonomy, and functional annotation

ORFs from each assembled contig were predicted using MetaGene [73] (http://metagene.cb.k.u-tokyo.ac.jp/). Predicted ORFs with a length of 100 bp or more were retrieved and translated into amino acid sequences using the NCBI translation table (http://www.ncbi.nlm.nih.gov/Taxonomy/taxonomyhome.html/index.cgi?chapter=tgencodes#SG1).

A nonredundant gene catalog was constructed using CD‐HIT [74] (version 4.6.1; http://www.bioinformatics.org/cd-hit/) with 90% sequence identity and 90% coverage. After QC, reads were mapped to the nonredundant gene catalog with 95% identity using SOAPaligner [75] (version 2.21; http://soap.genomics.org.cn/), and gene abundance in each sample was evaluated.

Representative sequences of nonredundant gene catalog were aligned to the NCBI NR database with a cutoff e‐value of 1e^−5^ using Diamond [76] (version 0.8.35; http://www.diamondsearch.org/index.php) for taxonomic annotations. A cluster of orthologous groups of proteins annotation for the representative sequences was performed using Diamond against eggNOG database with a cutoff e‐value of 1e^−5^. KEGG annotation was conducted using Diamond against the KEGG database (http://www.genome.jp/keeg/) with an e‐value cutoff of 1e^−5^.

The diversity of the microbial community was calculated using Explicet software [77]. Alpha diversity was determined using the minimum library size as the default with 1000‐bootstrap resampling. Beta diversity was assessed using the Bray‐Curtis similarity distance metric. R programming was used to visualize metagenomic and metabolic data.

### Fecal metabolite extraction and UHPLC‐MS/MS analysis

Metabolites were extracted from each feces sample (50 mg) using a 400 μL methanol:water (4:1, v/v) solution, with 0.3 mg/mL l‐2‐chlorophenylalanin as the internal standard. The mixture was allowed to settle at −10°C and treated using high‐throughput tissue crusher Wonbio‐96c (Shanghai Wanbo Biotechnology Co. Ltd.) at 50 Hz for 6 min, followed by ultrasound at 40 kHz for 30 min at 5°C. Samples were placed at −20°C for 30 min to precipitate proteins. After centrifugation at 13,000*g* and 4°C for 15 min, the supernatant was carefully transferred to sample vials for LC‐MS/MS analysis. Chromatographic separation of the metabolites was performed using a Thermo ultra‐high‐performance liquid chromatography (UHPLC) system equipped with an ACQUITY BEH C18 column (100 × 2.1 mm i.d., 1.7 μm; Waters). As part of system conditioning and the QC process, a pooled QC sample was prepared by mixing equal volumes of all of the samples. The QC samples were disposed and tested in the same manner as the analytic samples. The use of QC samples helped to represent the whole sample set, which would be injected at regular intervals (every 10 samples) to monitor the stability of the analysis. Chromatographic separation of the metabolites was performed on a Thermo UHPLC system equipped with an electrospray ionization (ESI) source operating in either positive or negative ion mode.

### Metabolomics data preprocessing and annotation

After ultra‐high performance liquid chromatography time‐of‐flight/mass spectrometry (UPLC‐TOF/MS) analyses, raw data were imported into the Progenesis QI 2.3 (Nonlinear Dynamics; Waters) for peak detection and alignment. The preprocessing results generated a data matrix consisting of the retention time, mass‐to‐charge ratio (*m*/*z*) values, and peak intensity. Metabolic features detected that at least 80% of samples in any setting were retained. After filtering, minimum metabolite values were imputed for specific samples in which the metabolite levels fell below the lower limit of quantitation; each metabolic feature was normalized by sum. The internal standard was used for data QC (reproducibility); metabolic features with the relative standard deviation of QC > 30% were discarded. Following the normalization procedures and imputation, statistical analysis was performed on log‐transformed data to identify significant differences in metabolite levels between comparable groups. Mass spectra of these metabolic features were identified using the accurate mass, MS/MS fragments spectra, and isotope ratio difference searched from reliable biochemical databases, such as HMDB (http://www.hmdb.ca/) and Metlin database (https://metlin.scripps.edu/). The mass tolerance between the measured *m*/*z* values and the exact mass of the components of interest was ±10 ppm. For metabolites with MS/MS confirmation, only MS/MS fragments with scores above 30 were considered to be confidently identified. Otherwise, metabolites were only given tentative assignments.

### Metabolomics statistical analysis

A multivariate statistical analysis was performed using ropls (Version 1.6.2; http://bioconductor.org/packages/release/bioc/html/ropls.html) R package from Bioconductor on Majorbio Cloud Platform (https://cloud.majorbio.com). Principal component analysis (PCA) using an unsupervised method was applied to obtain an overview of the metabolic data, and general clustering, trends, or outliers were visualized. All the metabolite variables were scaled to unit‐variances before conducting the PCA. Orthogonal partial least‐squares discriminate analysis (OPLS‐DA) was used for statistical analysis to determine global metabolic changes between comparable groups. All of the metabolite variables were scaled to Pareto Scaling before conducting the OPLS‐DA. The model validity was evaluated from model parameters R2 and Q2, which provide information regarding the interpretability and predictability, respectively, of the model and avoid the risk of overfitting. Variable importance in the projection (VIP) was calculated for the OPLS‐DA model. *P *values were estimated using paired Student's *t* test on single‐dimensional statistical analysis. Statistically significant differences among groups were selected based on VIP value >1 and *P* < 0.05. A total of 24,180 differential peaks were selected, including 12,430 peaks in ESI+ and 11,750 peaks in ESI−. Differential metabolites among the two groups were summarized and mapped into their biochemical pathways through metabolic enrichment and pathway analysis based on the KEGG database search (KEGG, http://www.genome.jp/kegg/). These metabolites can be classified according to the pathways they are involved in or the functions they performed. Enrichment analysis was used to analyze a group of metabolites in a function node based on its appearance. The principle was that the annotation analysis of a single metabolite develops into an annotation analysis of a group of metabolites. The Python package scipy.stats (https://docs.scipy.org/doc/scipy/) was used to identify statistically significantly enriched pathways using Fisher's exact test.

### Statistics analysis

Statistical analysis was performed using R (https://cran.r-project.org/). An unpaired two‐tailed *t* test or Fisher's least significant difference test was conducted to determine significant differences between different treatments. The data were visualized using imageGP [78].

## CONFLICTS OF INTEREST

The authors declare no conflicts of interest.

## AUTHOR CONTRIBUTIONS

Xiaofang Li initiated the concept. Xin Zheng designed the animal toxicology experiments and analyzed the data. Likun Wang performed the high‐throughput sequencing data analysis. Linhao You performed animal experiments and physiological tests. Yong‐Xin Liu contributed to bioinformatics analysis. Siyu Tian performed the cell experiments. Wenjun Li performed the cadmium content determination. Xin Zheng, Xiaofang Li, and Likun Wang drafted the manuscript. All authors revised and approved the manuscript.

## Supporting information

Supporting Information.

Supporting Information.

## Data Availability

All the high‐throughput sequencing data have been deposited in GenBank under submission number SUB9883487 (metagenome sequencing) and BioProject accession number PRJNA739823. The figures related tables and scripts were deposited in https://github.com/uqxli12/imeta-dietary-licorice.
