# Correction

**DOI:** 10.1002/imt2.67

**Published:** 2022-11-23

In Zheng et al. [1], following ethics statement should have been added.

The ethics application (No. 2019SC05) was approved by the Research Ethics Committee of Hebei Normal University.

The authors wish to apologize for the error.
