# Supplementary material for: Dietary licorice enhances in vivo cadmium detoxification and modulates gut microbial metabolism in mice
Source: Imeta. 2022 Mar 10;1(1):e7. doi: 10.1002/imt2.7 (PMC10989944; doi:10.1002/imt2.7)
Supplement: Supplementary file 1 — Supporting Information. [file IMT2-1-e7-s001.docx]

Supplementary Material for

**Dietary licorice enhances *in vivo* cadmium detoxification and modulates gut microbial metabolism in mice**

Xin Zheng, Likun Wang, Linhao You*, Yong-Xin Liu, Michael Cohen, Siyu Tian, Wenjun Li, Xiaofang Li*

Correspondence author: Xiaofang Li ([xfli@sjziam.ac.cn](mailto:xfli@sjziam.ac.cn)), Linhao You ([youlh2009@163.com](mailto:youlh2009@163.com))

**
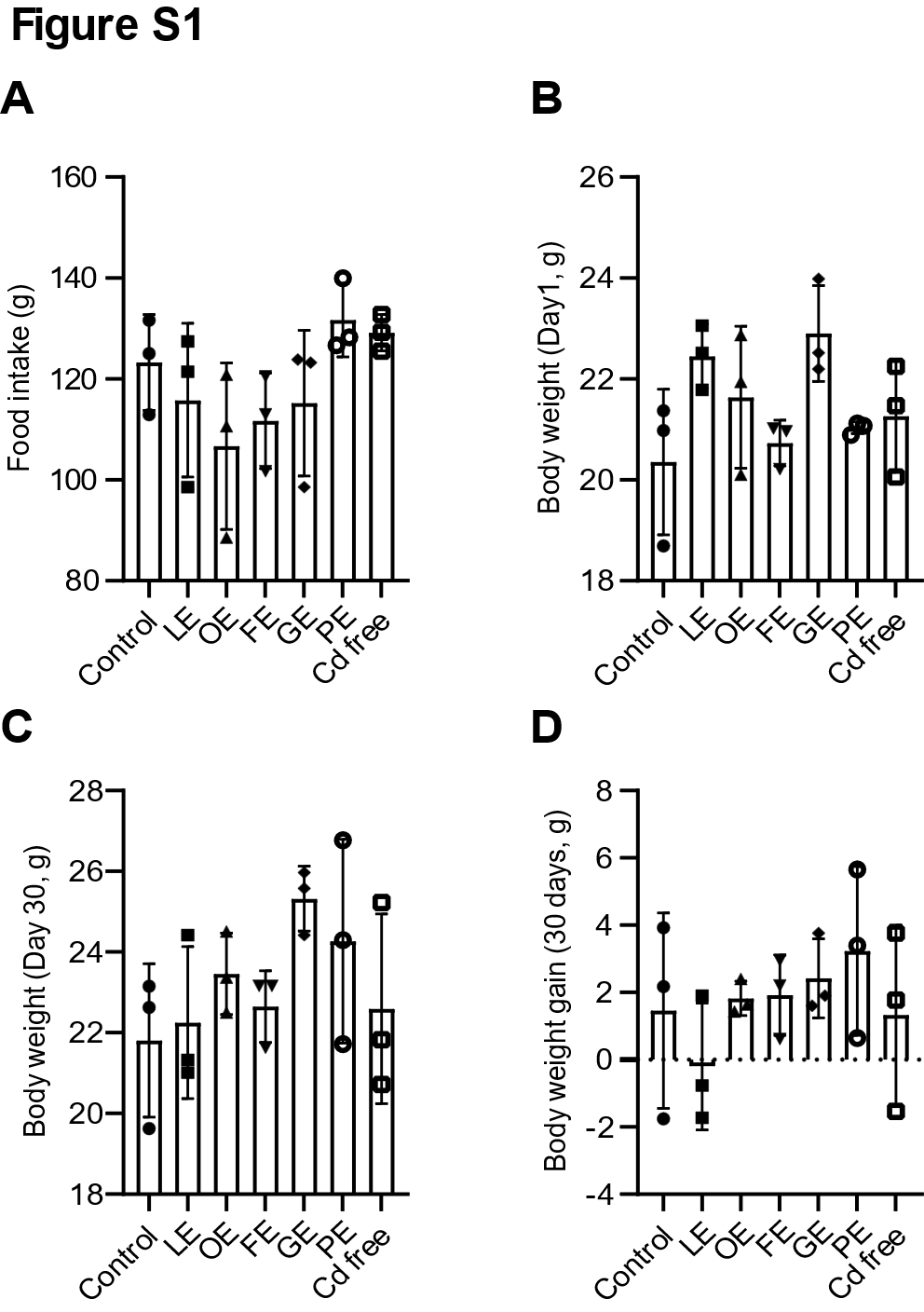
**

**Figure S1.** The food intake and body weight change after one-month different dietary interventions. LE, licorice extract; OE, onion extract; FE, fennel extract; GE, ginger extract; PE, pepper extract.

**
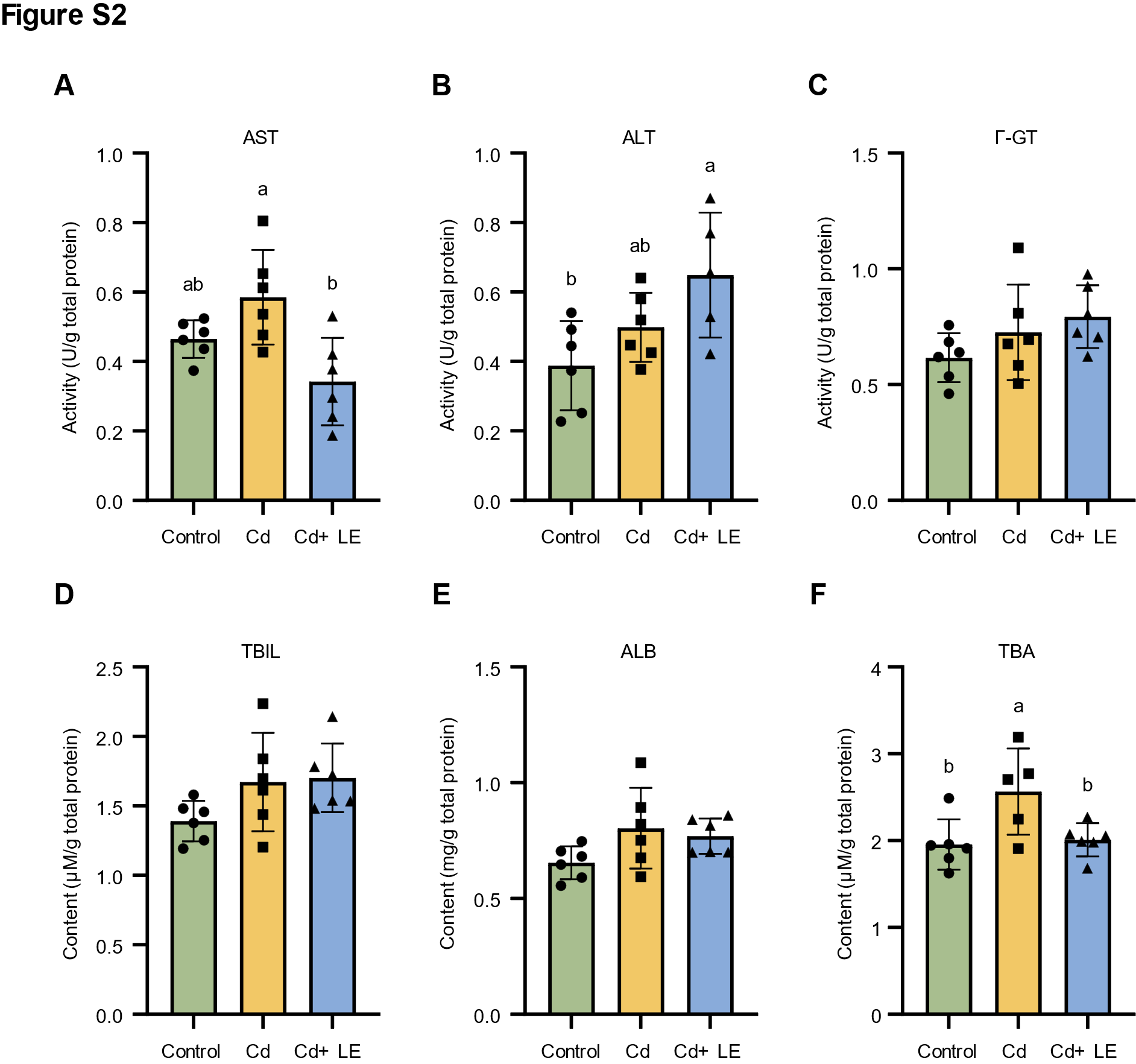
**

**Figure S2.** Hepatic function-related indices after one-month dietary licorice intervention. AST, aspartate aminotransferase; ALT, alanine aminotransferase; Γ-GT, Γ-glutamyltranspeptidase; TBIL, total bilirubin; ALB, albumin; TBA, total bile acid.

**
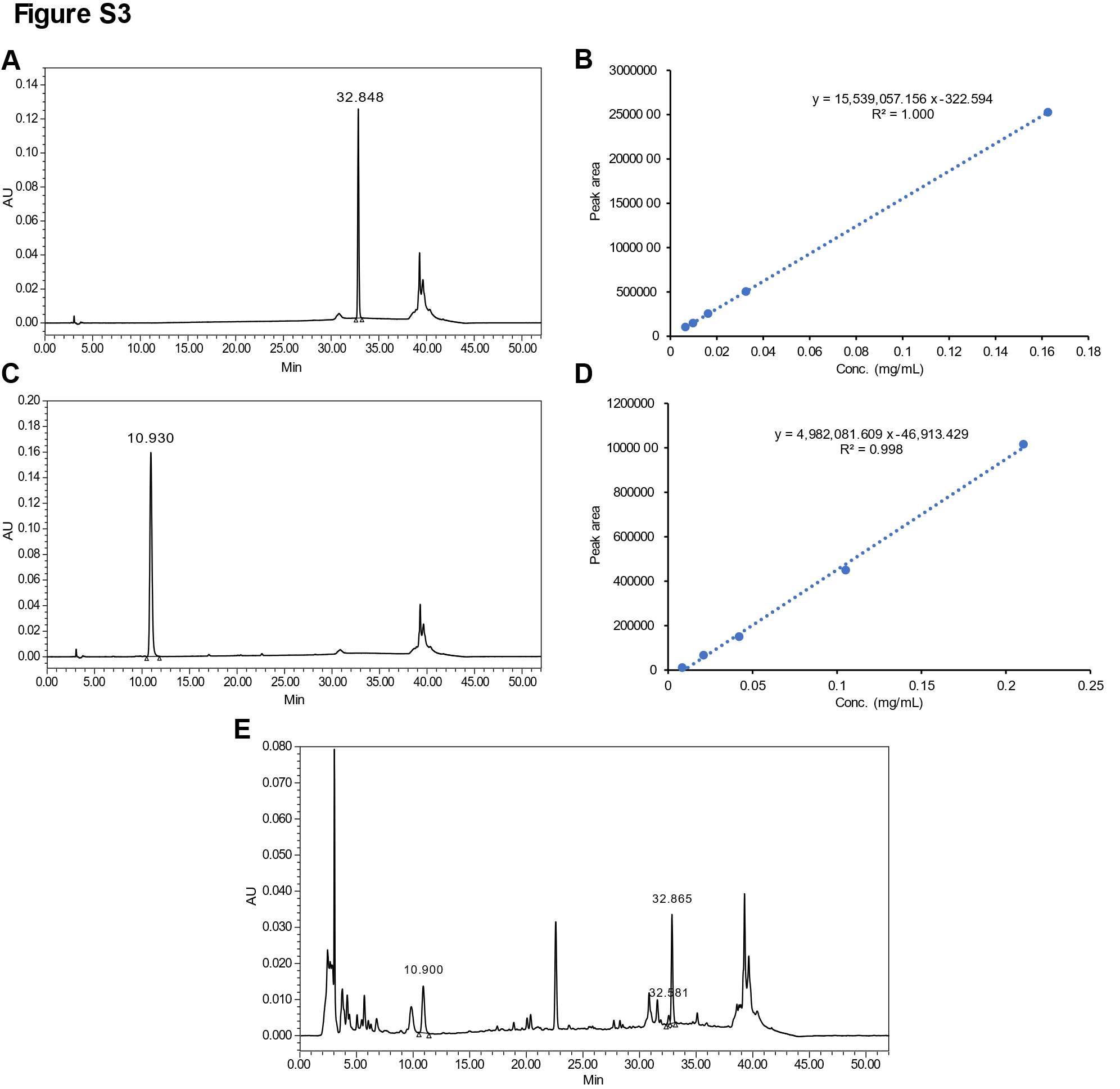
**

**Figure S3.** HPLC quantification of glycyrrhizin and liquiritin in the licorice extract powder used in this study. **(A** and **B)** Chromatographic peak and standard curve of standard glycyrrhizin. **(C** and **D)** Chromatographic peak and standard curve of standard liquiritin. **(E)** Chromatographic peak of licorice extract powder used in this study.

**
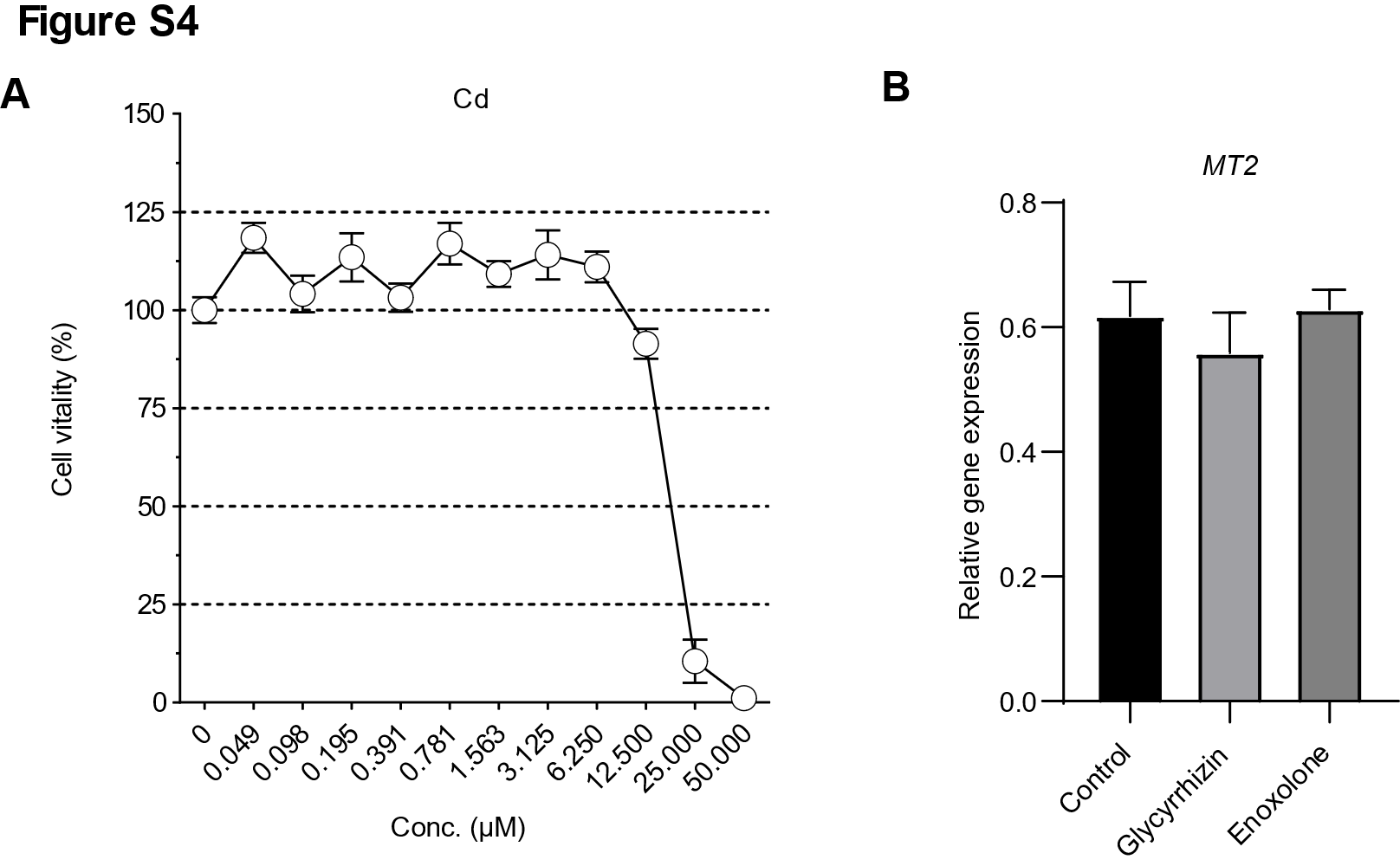
**

**Figure S4. (A)** Cell viability test by using mice cell line AML12 against a concentration gradient of cadmium (n=3). **(B)** The cadmium-exposed AML12 cells were treated with licorice extract, glycyrrhizin and enoxolone for 12 h, respectively, and expression level of *Mt2* gene (n=3) was determined by qPCR analysis**.**

**
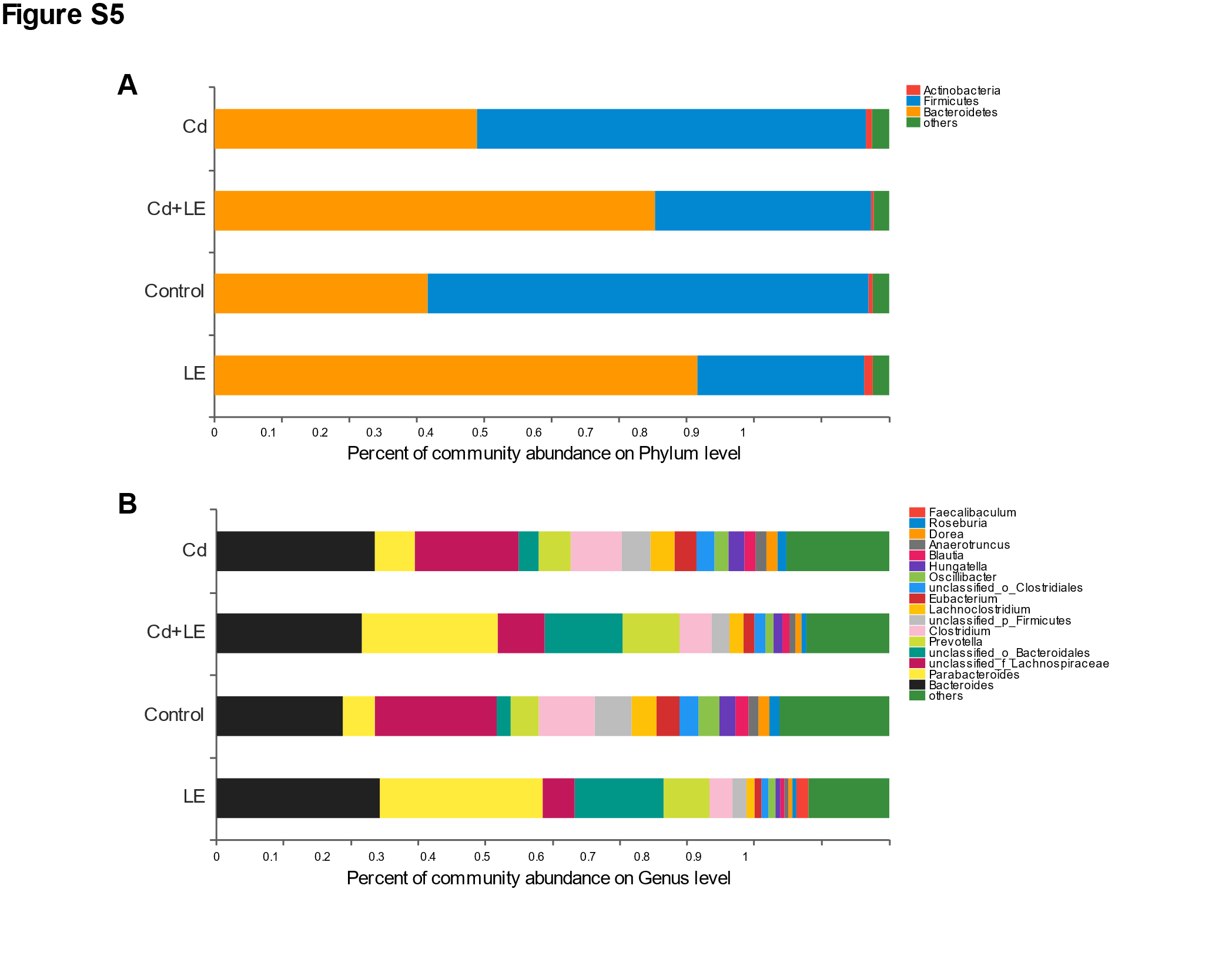
**

**Figure S5.** Community barplot analysis on Phylum level **(A)** and Genus level **(B)** in the metagenomic sequencing.

**
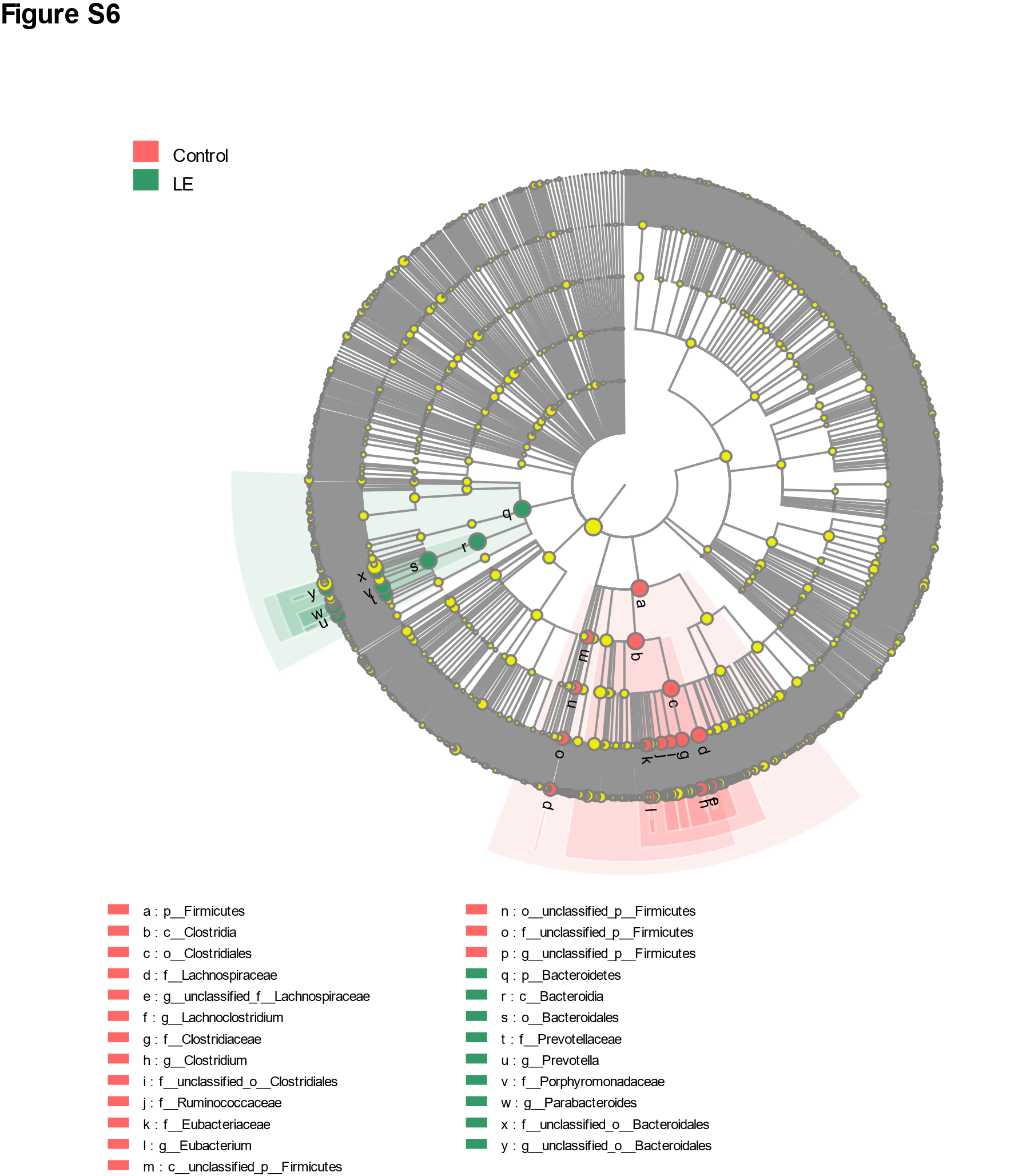
**

**Figure S6.** LEfSe test showing the distinctive gut microbes (LDA > 4) in Control and LE groups.

**
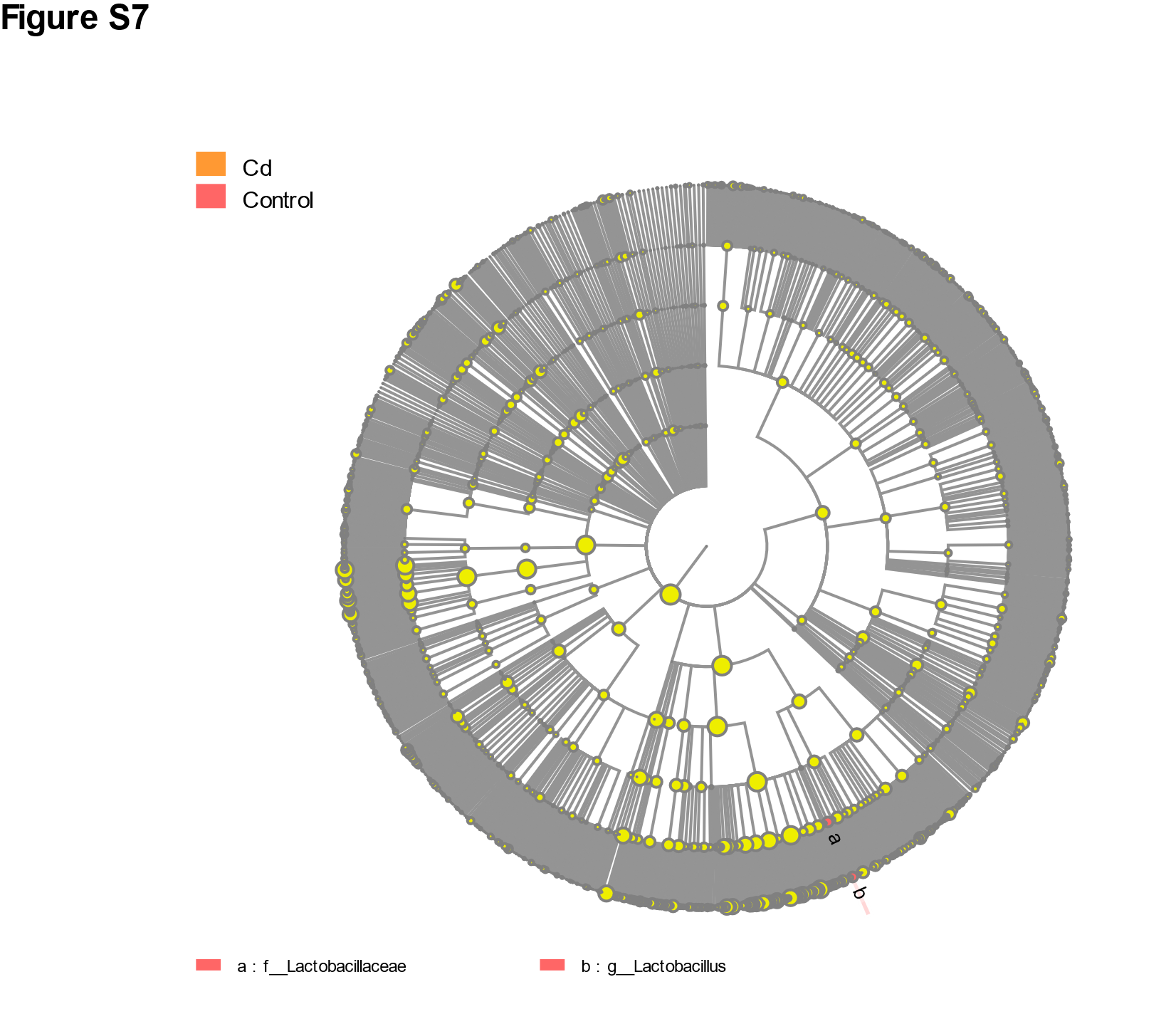
**

**Figure S7.** LEfSe test showing the distinctive gut microbes (LDA > 2) in Control and Cd groups.

**
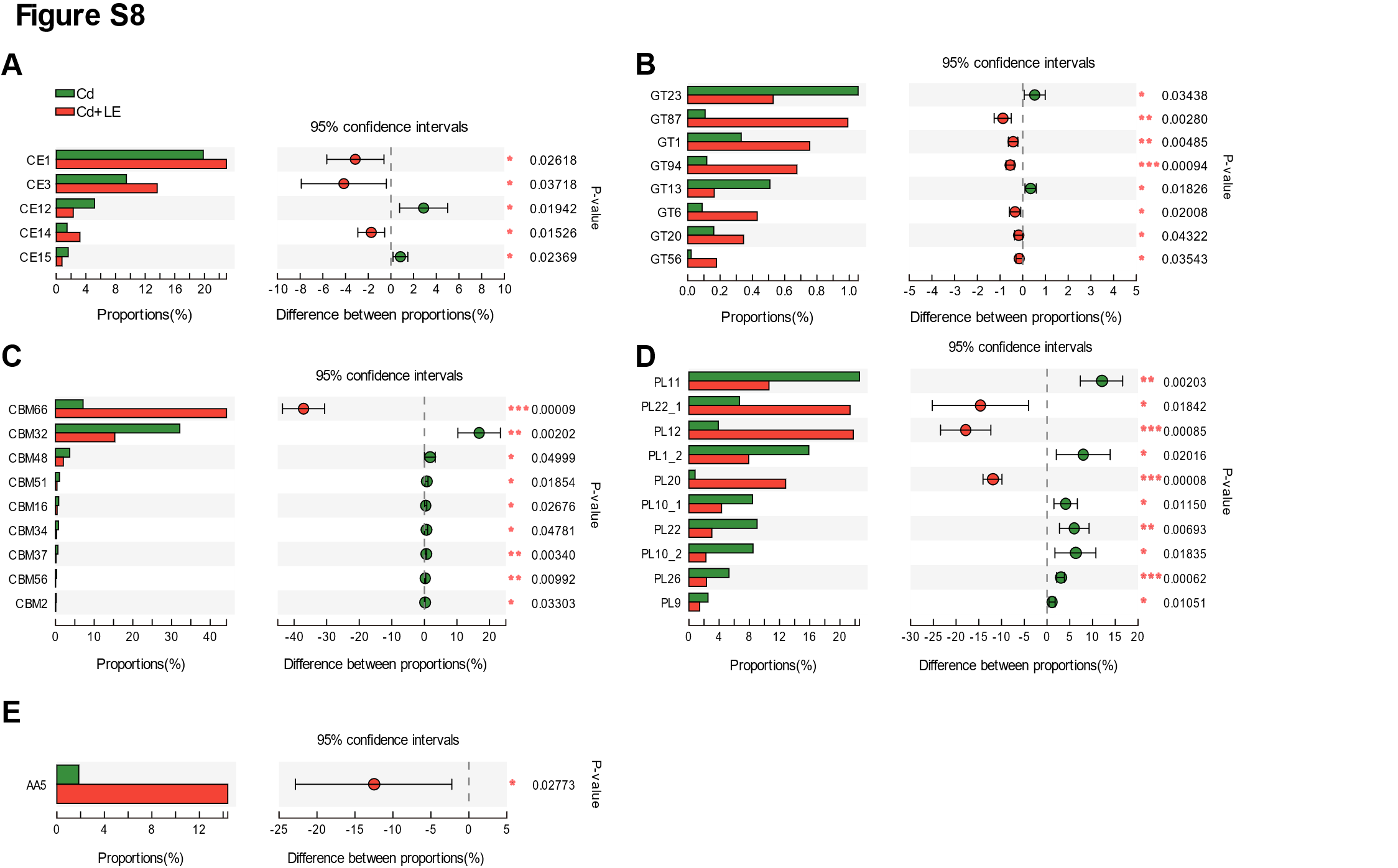
**

**Figure S8.** The carbohydrate-active enzyme families with significant differential abundance between Cd and Cd+LE groups. **(A)** Carbohydrate esterases families. **(B)** Glycosyl transferases families. **(C)** Carbohydrate-binding modules families. **(D)** Polysaccharide lyases families. **(E)** Auxiliary activities families.


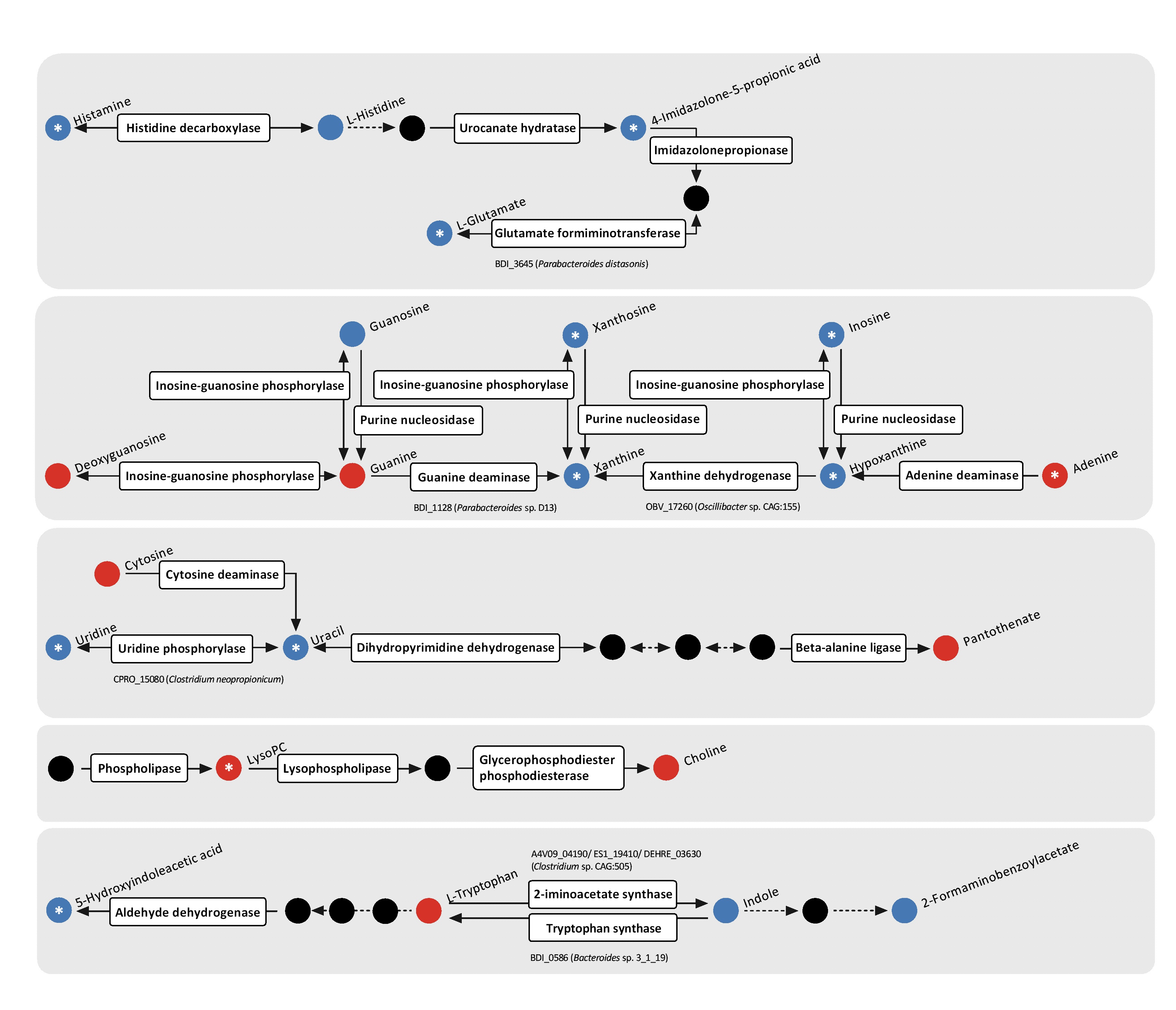


**Figure S9.** Faeces metabolites and potential metabolic pathways of gut microbiota associated with the dietary licorice intervention. The pathways were constructed based on the KEGG metabolic maps. Metabolites are indicated as red (enriched in the Cd + LE group), blue (enriched in the Cd group), or black (none detected) balls. The dashed arrow indicates the potential metabolic process without detection of relevant enzyme-encoding genes. Relative abundance of the involved metabolites (mean ± SD, *n* = 6, **P* < 0.05) are shown.
